# Virtual Screening–Guided Discovery of a Selective TRPV1 Pentapeptide Inhibitor with Topical Anti-Allergic Efficacy

**DOI:** 10.3390/cells15010079

**Published:** 2026-01-03

**Authors:** Lulu Liu, Wenqian Hou, Qinyi He, Fuchu Yuan, Changrun Guo, Ruxia Liu, Biao Huang, Atikan Wubulikasimu, Mingqiang Rong

**Affiliations:** 1 The National & Local Joint Engineering Laboratory of Animal Peptide Drug Development, Peptide and Small Molecule Drug R&D Platform of Furong Laboratory, College of Life Sciences, Hunan Normal University, Changsha 410081, China; liull2025@hunnu.edu.cn (L.L.); houwenqian@hunnu.edu.cn (W.H.);; 2 Suixian Public Inspection and Testing Center, Suizhou 441300, China; 3 Frontiers Medical Center, Tianfu Jincheng Laboratory, Chengdu 610212, China; 4 School of Basic Medicine and Clinical Pharmacy, China Pharmaceutical University, Nanjing 210009, China

**Keywords:** structure-based virtual screening, pentapeptide inhibitors, TRPV1, cutaneous allergy, topical therapy

## Abstract

Transient receptor potential vanilloid 1 (TRPV1) channels are critical mediators of cutaneous allergic inflammation, contributing to pruritus, erythema, and hypersensitivity in allergic skin disorders. Despite their therapeutic potential, clinically available TRPV1 inhibitors remain limited, leaving effective treatment options lacking. Here, we focused on a self-constructed virtual pentapeptide library and identified a highly selective TRPV1 inhibitor that demonstrated pronounced anti-allergic effects in human skin assays. Through structure-based virtual screening of approximately 200,000 peptide conformations, five candidate pentapeptides, especially P5 (DQKNC), exhibited the inhibition. Electrophysiological recordings showed that P5 inhibited TRPV1 currents with nanomolar potency, while exhibiting negligible effects on major cardiac and neuronal ion channels, highlighting its favorable selectivity and safety profile. In capsaicin-induced human skin hypersensitivity tests, topical P5 significantly reduced burning pain, erythema, and pruritus, with simultaneous application providing the most robust relief. These findings reveal a short peptide with strong TRPV1 selectivity and demonstrable efficacy in alleviating skin inflammation and allergic responses, supporting the notion that rationally designed pentapeptides may represent promising topical therapeutics for allergic skin disorders.

## 1. Introduction

As part of the body’s defense mechanisms, Transient Receptor Potential Vanilloid 1 (TRPV1) channels function as a primary sensor for thermal injury by triggering avoidance behaviors that reduce tissue damage [1,2,3]. Beyond sensing physical injury, TRPV1 also responds to chemical and immunological stimuli in the skin, linking environmental triggers to local inflammatory and allergic responses [4,5]. At the same time, TRPV1 acts as a key receptor in skin allergic responses, mediating neurogenic inflammation and directly contributing to the initiation and amplification of cutaneous inflammatory reactions [5,6]. Given its high sensitivity to mechanical and thermal stimuli as well as neurogenic inflammation, the development of TRPV1 inhibitors holds considerable promise for anti-inflammatory interventions [7,8].

TRPV1 inhibitors, by blocking Ca^2+^ influx in sensory neurons, reduce the excitability of primary afferents and alleviate pain, burning sensations, and inflammatory responses [9]. Moreover, TRPV1 inhibitors have demonstrated potential efficacy in conditions such as cutaneous inflammation [10,11]. Several TRPV1 inhibitors have advanced into clinical trials; for instance, Asivatrep (PAC-14028) has successfully completed phase III clinical evaluation, showing significant clinical benefits in relieving pruritus and improving atopic dermatitis–related allergic skin symptoms [12,13]. Whereas GSK’s SB-705498 failed to effectively alleviate both histamine-dependent and histamine-independent pruritus in a phase I trial [14,15]. Thus, TRPV1 inhibitors remain in the developmental stage, with no systemically approved therapeutic options available to date.

Furthermore, conventional small-molecule inhibitor development is constrained by high costs, low efficiency, and lengthy development cycles. The application of virtual screening (VS) provides an effective strategy to overcome these limitations, offering not only savings in experimental resources and time but also improved environmental sustainability [16,17]. VS, including ligand- and structure-based methods (LBVS and SBVS), especially when combined with artificial intelligence and deep learning (e.g., GNNs), can greatly improve screening efficiency and prediction accuracy [18,19]. In this study, we applied an SBVS approach to construct a virtual pentapeptide library and identified five candidate pentapeptides (P1–P5) as potential TRPV1 inhibitors. Functional assays demonstrated that candidate peptide P5 exhibits high TRPV1 selectivity and significantly attenuates capsaicin-induced cutaneous anaphylaxis, underscoring its therapeutic potential in allergic skin inflammation and localized inflammatory disorders.

## 2. Materials and Methods

### 2.1. Construction of a Virtual Pentapeptide Database

A virtual pentapeptide library was constructed using Python (version 3.12), by randomly combining the 20 standard amino acids—alanine (A), cysteine (C), aspartic acid (D), glutamic acid (E), phenylalanine (F), glycine (G), histidine (H), isoleucine (I), lysine (K), leucine (L), methionine (M), asparagine (N), proline (P), glutamine (Q), arginine (R), serine (S), threonine (T), valine (V), tryptophan (W), and tyrosine (Y). Each pentapeptide was saved in FASTA format with a unique identifier, resulting in a library of 205 pentapeptides. The library was subsequently batch-converted from FASTA to SMILES format using RDKit, generating a 2D structural library. Three-dimensional conformations of the pentapeptides were then generated using the LigPrep module in Schrödinger (version 2021).

#### Molecular Docking

The crystal structure of the rat TRPV1 channel (PDB ID: 5IS0) was obtained from the Protein Data Bank (PDB). Protein preparation was performed using the Protein Preparation Wizard module in Schrödinger to optimize the rTRPV1 structure. The Receptor Grid Generation function in Glide was employed to define the binding site, with custom coordinates centered on residues Gly643, Met644, Gly645, Asp646, Leu647, Glu648, Phe649, and Thr650. A docking grid box was generated with an outer box size of 20 Å and an inner box size of 10 Å.

Prepared pentapeptide ligands were docked into the receptor grid using the Glide Ligand Docking workflow. The receptor grid file (“GRIDFILE”) and ligand file (“LIGANDFILE”) were specified as input. High-throughput virtual screening (HTVS) was first applied, and the top 30% scoring peptides were further evaluated using the standard precision (SP) scoring function. The maximum number of poses to be recorded (“NREPORT”) was set to 5000. Output options were configured with “WRITE_CSV” set to “True” for tabular data export and “WRITE_RES_INTERACTION” set to “True” to generate residue-level interaction details.

For ADMET-related prioritization, the top 20 pentapeptide candidates ranked by docking score were further evaluated using the SwissADME online tool. Key predicted pharmacokinetic properties, including lipophilicity (Consensus LogP), aqueous solubility (ESOL LogS), gastrointestinal absorption, and blood–brain barrier permeability, were assessed and used in combination with docking scores to prioritize candidate peptides for subsequent analysis.

### 2.2. Peptide Synthesis and Purification

Linear peptides were synthesized using standard Fmoc solid-phase peptide synthesis (SPPS) on a resin, with amino acids sequentially coupled from the C-terminus to the N-terminus. Crude peptides were dissolved in ultrapure water, centrifuged at 10,000× *g* for 10 min, and filtered through a 0.22 μm membrane. Purification was performed by reversed-phase high-performance liquid chromatography (RP-HPLC), with target peaks identified by mass spectrometry. Collected fractions of the target peptide were lyophilized and stored at −20 °C until use.

### 2.3. Cell Culture and Transient Transfection

The plasmids encoding rTRPV1, Nav1.5, Nav1.8, Kv4.2, and hERG were sourced from our laboratory. The TRPV3 plasmid was commercially obtained from Wuhan MiaoLing Biotechnology Co., Ltd. (Wuhan, China) HEK293 cells, purchased from the Kunming Cell Bank, Kunming Institute of Zoology, Chinese Academy of Sciences, were cultured in Dulbecco’s modified Eagle’s medium supplemented with 10% fetal bovine serum and 1% penicillin/streptomycin at 37 °C in 5% CO_2_. These cells were transiently transfected with the channel-expressing plasmid carrying a green fluorescent protein tag using Lipofectamine 2000 transfection reagent. Patch-clamp recordings were conducted 24 h post-transfection.

### 2.4. Electrophysiology

Whole-cell recordings were conducted at room temperature using an EPC10 amplifier (HEKA Elektronik, Lambrecht/Pfalz, Germany) and PatchMaster software (v2x90.3). Patch pipettes were fabricated from borosilicate glass and fire-polished to a resistance of approximately 4 MΩ. For TRPV1 and mTRPV3 channels recordings, a holding potential of −60 mV was applied. The pipette solution contained 140 mM CsCl, 5 mM EGTA, and 10 mM HEPES, adjusted to pH 7.4 with CsOH. The bath solution contained 140 mM NaCl, 5 mM KCl, 3 mM EGTA, and 10 mM HEPES, adjusted to pH 7.4 with NaOH. For Kv4.2 channel recordings, cells were held at −80 mV, and currents were evoked upon depolarization to 0 mV. The pipette solution contained 140 mM KCl, 2.5 mM MgCl_2_, 10 mM HEPES, and 10 mM EGTA, adjusted to pH 7.4 with KOH. The bath solution contained 140 mM NaCl, 5 mM KCl, 2 mM CaCl_2_, 1 mM MgCl_2_, 10 mM HEPES, and 10 mM glucose, adjusted to pH 7.4 with NaOH. For hERG channel recordings, cells were held at −80 mV, depolarized to +60 mV for 2 s, and then stepped to −40 mV for 2 s to elicit hERG currents. The pipette and bath solutions were identical to those used for Kv4.2 recordings. For Nav1.5 and Nav1.7 channel recordings, cells were held at −90 mV, and currents were evoked upon depolarization to 0 mV. The pipette solution contained 140 mM CsF, 1 mM EGTA, 10 mM NaCl, and 10 mM HEPES, adjusted to pH 7.4 with CsOH. The bath solution contained 140 mM NaCl, 5 mM KCl, 2 mM CaCl_2_, 1 mM MgCl_2_, 10 mM HEPES, and 10 mM glucose, adjusted to pH 7.4 with NaOH.

Transfected channel cells were positioned at the perfusion tube outlet, and capsaicin (Cap, TRPV1 agonist) or 2-aminoethoxydiphenyl borate (APB, mTRPV3 agonist) were delivered via a gravity-driven system RSC-200 (Bio-Logic Science Instruments, Seyssinet-Pariset, France) with nine separate tubes, while currents were recorded.

### 2.5. Human Skin Patch Test

To assess the efficacy of P5 in alleviating skin erythema, pruritus, and pain, a capsaicin-induced skin irritation patch model was established. Twelve healthy volunteers (male and female, aged 18–40 years) with no history of skin disorders were recruited after providing informed consent. The experiment was conducted using a double-blind design to minimize bias in both treatment administration and outcome evaluation. Experiments were performed simultaneously on the volar surfaces of both forearms using two models: co-administration and pre-administration, with the co-administration model conducted on the left volar forearm and the pre-administration model conducted on the right volar forearm. In the co-administration model, three treatments were prepared: Control: 0.8 mM capsaicin (Cap) + saline; P5 group: 0.8 mM Cap + 5 mM P5; Positive control: 0.8 mM Cap + 0.1 mM capsazepine (CPZ). Each 20 μL solution was applied to a filter paper disc in a patch device and attached to the left volar forearm. Photographs were taken at 1 h and 3 h post-application. In the pre-administration model, filter paper discs were preloaded with 10 μL of saline, 5 mM P5, or 0.1 mM CPZ and applied to the right volar forearm for 1 h. Subsequently, 10 μL of 0.8 mM Cap was added to each disc simultaneously. Photographs were recorded at 1 h and 3 h after Cap application. Each volunteer received all three treatments at different skin sites, rather than being divided into separate treatment groups. Each 20 μL solution was applied to a filter paper disc in a patch device and attached to the left volar forearm. The filter paper discs used in the skin patch devices measured 1 cm × 1 cm, with a maximum liquid absorption capacity of 25 μL, and were backed with medical-grade waterproof adhesive tape. The filter paper discs corresponding to the three treatment conditions were evenly distributed along the volar surface of the forearm, with an inter-patch distance of approximately 1–3 cm, to avoid overlap or interference between adjacent treatment sites.

Volunteers self-reported skin perception using two scoring systems. The Sensory Score ranged from one to four, with one indicating no sensation, two indicating mild sensation such as itch, heat, or pain, three indicating moderate sensation including stinging, heat, or itch, and four indicating severe burning, pain, or itch. The Sensation Time Score was based on the time to onset of discomfort after compound application. In the co-administration model, timing started immediately after compound application, while in the pre-administration model, timing started after capsaicin addition. Scores were assigned as follows: a score of one corresponded to zero to half an hour, two to half an hour to one hour, three to one to one and a half hours, four to one and a half to two hours, five to two to two and a half hours, and six to more than two and a half hours.

All scoring data were analyzed using one-way ANOVA in GraphPad Prism (version 8.0.1). Statistical significance was defined at α = 0.05 and indicated as *p* ≤ 0.05 (*), *p* ≤ 0.01 (**), *p* ≤ 0.001 (***). Data are presented as mean ± S.D.

### 2.6. Statistics and Reproducibility

Electrophysiological data were analyzed using Igor Pro (version 6.37) and Prism GraphPad (version 8.0.1). All the results are presented as mean ± SEM.

The concentration-response relationships of the pentapeptide at TRPV1, TRPV3, Nav1.5, Nav1.7, Kv4.2, and hERG were fitted to the Hill equation.

$$\frac{I_{x}}{I_{max}}=1-\frac{{\left[x\right]}^{n}}{{\left[x\right]}^{n}+IC_{50}^{n}}$$

where *I_x_* represents the difference between the steady-state TRPV1 and Kv4.2 channels current and the leaking current in the presence of concentration [*x*]; [*x*] represents the concentration of pentapeptide; *I_max_* represents the difference between the maximal current amplitude and the leaking current; *IC*_50_ represents the concentration of pentapeptide at which inhibition is half-maximal; *n* represents the Hill coefficient.

## 3. Results

### 3.1. Virtual Screening of rTRPV1 Pentapeptide Inhibitors

**Figure 1:**
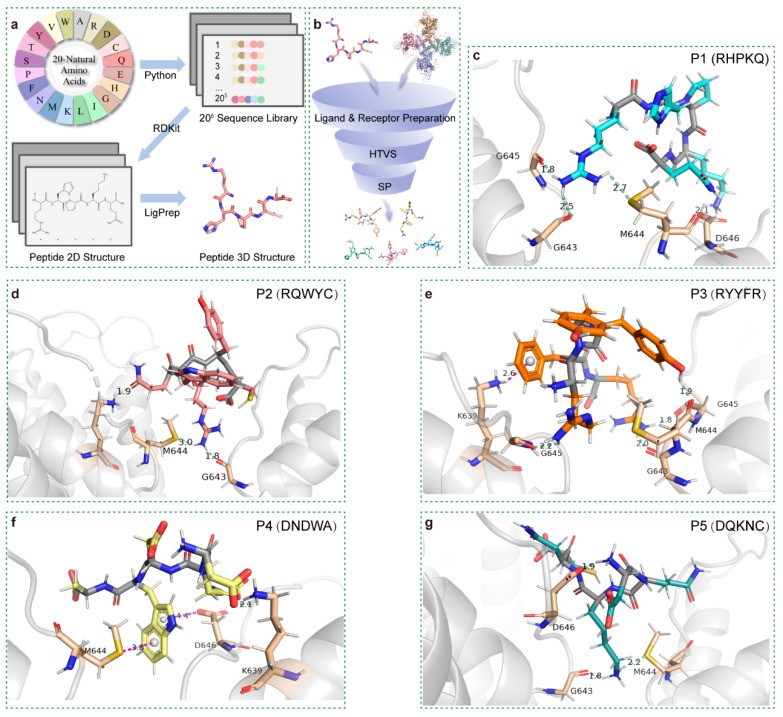
Construction of a virtual pentapeptide library and binding modes with the TRPV1 protein. (**a**) Schematic workflow of the virtual pentapeptide library. Twenty amino acids, represented by single-letter codes, were randomly combined into pentapeptide sequences using a Python script. The sequences were converted into 2D molecular structures with RDKit and further processed in the LigPrep module to generate energy-minimized 3D conformations. (**b**) Hierarchical molecular docking–based screening workflow using the Glide module. HTVS was first used for rapid preliminary screening, followed by standard precision (SP) docking of top-ranked HTVS candidates for more refined evaluation. (**c**–**g**) Representative binding modes of the five selected pentapeptides (P1–P5) with the TRPV1 protein. Side chains of P1–P5 are displayed as stick models in cyan, salmon, orange, pale yellow, and light teal, respectively; backbones are shown in gray. Nitrogen atoms are colored blue, oxygen atoms red, and sulfur atoms yellow. Interacting receptor residues are shown as wheat-colored sticks with the same atom color scheme. Hydrogen bonds are depicted in pale green, and hydrophobic interactions in magenta.

A virtual library of 20^5^ pentapeptides was successfully established for rTRPV1 inhibitor screening by generating sequence permutations of the 20 standard amino acids. The virtual library was subsequently converted into three-dimensional structures using RDKit and Schrödinger LigPrep (Figure 1a). This process built a library with approximately 200,000 three-dimensional peptide conformations. High-throughput virtual screening (HTVS) was performed by docking these preprocessed peptides against the TRPV1 receptor, followed by standard precision (SP) docking of the top 30% ranked peptides (Figure 1b). For benchmarking and validation of the docking pipeline, the known TRPV1 antagonist capsazepine was docked under identical conditions as a reference ligand (Table S2). Based on a combined assessment of docking scores and predicted ADMET-related properties (Table S3), five candidate pentapeptides were identified with docking scores below −10 (Table S1): P1 (RHPKQ), P2 (RQWYC), P3 (RYYFR), P4 (DNDWA), and P5 (DQKNC). TRPV1 consists of six transmembrane α-helices (S1–S6), which form a voltage sensor–like domain (VSLD, S1–S4) and a pore domain (S5–S6) connected via the S4–S5 linker [20,21].All five candidates bound to the outer pore region formed by the S5–S6 segments. Considering the structural constraints of short pentapeptides, we targeted a predicted binding pocket near the selectivity filter (Gly643–Met644–Gly645) as the most plausible site for pore blockade [22]. Peptides P1–P3 inhibit TRPV1 currents by inserting a lysine side chain into the pore, where it forms hydrogen bonds with one or more residues within the upper segment of the pore-region selectivity filter (G643–M644–G645), thereby blocking ion conduction (Figure 1c–e). Similarly, P4 and P5 interacted with the pore by inserting tryptophan (Figure 1f) and lysine side chains (Figure 1g), respectively. Other docking poses yielded highly similar binding patterns and residue engagement (Figure S1). Collectively, these binding modes revealed a shared mechanism: a long-chain amino acid residue inserts into the narrow pore, forming hydrogen bonds with residues of the selectivity filter or adjacent sites, while other residues contribute to stabilizing peptide binding above the filter region.

### 3.2. P3 and P5 Significantly Inhibit rTRPV1

**Figure 2:**
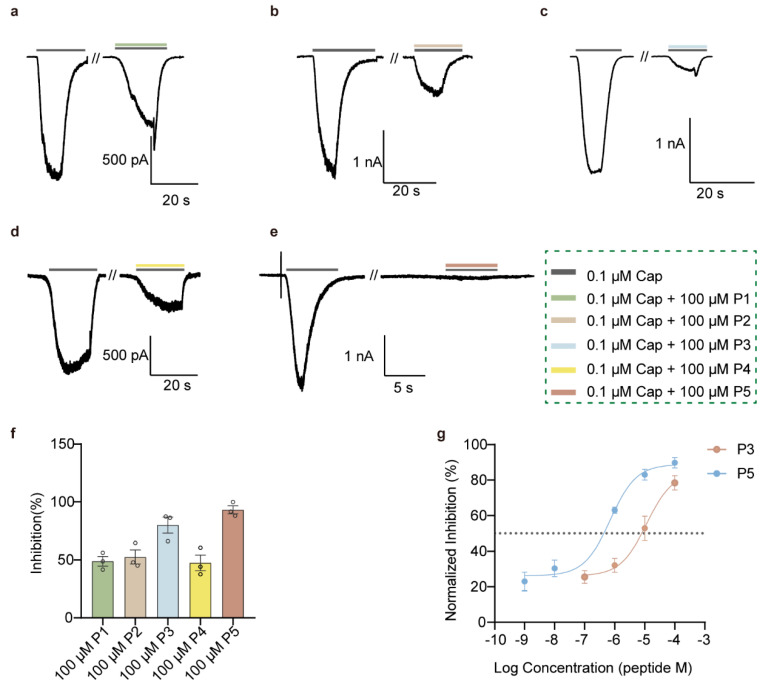
Inhibitory activity of candidate peptide inhibitors on TRPV1 channels. (**a**–**e**) Whole-cell currents showing the inhibitory effects of 100 μM P1–P5 on hTRPV1 channels. (**f**) Bar graph summarizing inhibition rates of hTRPV1 currents by 100 μM P1–P5 (*n* = 3 per group). (**g**) Concentration–response curves of hTRPV1 inhibition by P3 and P5 fitted to the Hill equation (*n* = 5 per group). All recordings were obtained at a holding potential of −80 mV. All statistical data are given as average ± SEM.

To investigate the function of the candidate pentapeptides, we synthesized linear peptides using Fmoc solid-phase peptide synthesis. MALDI-TOF mass spectrometry analysis demonstrated that the molecular weights of all five peptides were consistent with their theoretical values (Figure S2). Given the gating properties of TRPV1, we applied capsaicin to maintain channel activation, and performed whole-cell recordings in HEK293 cells expressing rTRPV1. All five peptides at 100 μM exhibited inhibitory effects of varying degrees on rTRPV1 currents (Figure 2a–e). The percentage inhibition was 48.88 ± 7.02% for P1, 52.61 ± 10.56% for P2, 80.01 ± 11.99% for P3, 47.5 ± 11.8% for P4, and 86.57 ± 6.05% for P5 (Figure 2f). Notably, P3 and P5 displayed the strongest inhibition, and subsequent IC_50_ determination revealed values of 11.44 μM for P3 and 0.71 μM for P5 (Figure 2g). Therefore, these results prompted us to evaluate the two candidate peptides on other key ion channels related to drug safety at the cellular level.

### 3.3. High-Selectivity rTRPV1 Inhibitor P5

**Figure 3:**
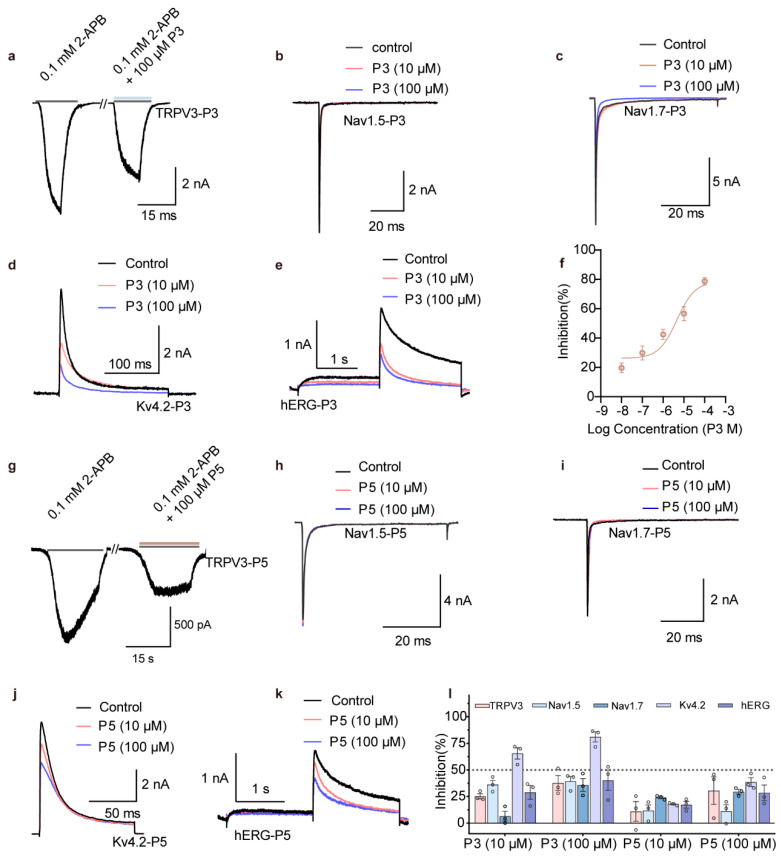
Selectivity profiles of peptide inhibitors P3 and P5 across TRPV1 and related ion channels. (**a**) Inhibitory effect of 100 μM P3 on open-state TRPV3 channels. (**b**–**e**) Whole-cell currents showing the inhibitory effects of 10 μM and 100 μM P3 on Nav1.5, Nav1.7, Kv4.2, and hERG channels. (**f**) Concentration–response curve of Kv4.2 inhibition by P3 fitted to the Hill equation (*n* = 5 per group). (**g**) Inhibitory effect of 100 μM P5 on open-state TRPV3 channels. (**h**–**k**) Whole-cell currents showing the inhibitory effects of 10 μM and 100 μM P5 on Nav1.5, Nav1.7, Kv4.2, and hERG channels. (**l**) Bar graph summarizing inhibition rates of TRPV3, Nav1.5, Nav1.7, Kv4.2, and hERG currents by 10 μM and 100 μM P3 and P5 (*n* = 3 per group). All statistical data are given as average ± SEM.

Nav1.5, Nav1.7, Kv4.2, and hERG are critical indicators in drug safety evaluation, as their inhibition can cause cardiac conduction block, bradycardia, arrhythmias, or altered nociception [23,24]. To investigate potential effects of P3 and P5, we constructed plasmids encoding these channels, transiently expressed them in HEK293 cells, and examined their functional responses using whole-cell patch-clamp recordings. The results showed that P3 produced modest inhibition of TRPV3, with a 37.7 ± 12.2% reduction at 100 μM (Figure 3a), but exhibited no significant inhibition of Nav1.5 or Nav1.7 at either 10 or 100 μM (Figure 3b,c). In contrast, P3 markedly suppressed Kv4.2 currents: at 100 μM it produced 81.1 ± 8.2% inhibition (Figure 3d), and concentration–response analysis yielded an IC_50_ of 6.50 μM (Figure 3f). Furthermore, P3 reduced hERG currents by 40.2 ± 16.4% at 100 μM (Figure 3e). For comparison, P5 also inhibited TRPV3, though to a lesser extent than TRPV1, achieving 30.1 ± 22.5% inhibition at 100 μM (Figure 3g). Importantly, P5 showed negligible effects on Nav1.5 and Nav1.7 at both 10 and 100 μM (Figure 3h,i), and its inhibition of Kv4.2 (38.6 ± 7.1%) and hERG (28.6 ± 12.8%) at 100 μM was substantially weaker than that of P3 (Figure 3j,k). Taken together, these results indicate that P5 potently inhibits TRPV1 and exhibits good selectivity against Nav1.5, Nav1.7, Kv4.2 and hERG at the cellular level (Figure 3i), thereby satisfying the basic safety requirements for topical human models.

### 3.4. P5 Alleviated Capsaicin-Induced Skin Hypersensitivity in Human Skin

**Figure 4:**
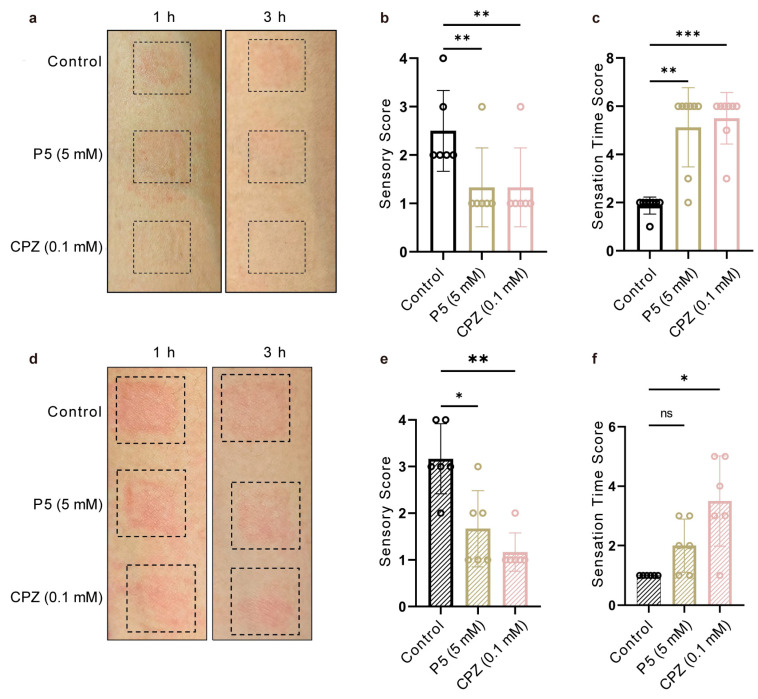
Evaluation of therapeutic effects in co-administration and pre-administration regimens in a human skin model. (**a**) Representative images of human dorsal skin from the control, P5-treated, and Capsazepine (CPZ) positive control groups at 1 and 3 h post-capsaicin challenge (co-administration regimen). (**b**) Sensory scores for the three groups in the co-administration regimen (*n* = 6; ns, not significant; * *p* < 0.05; ** *p* < 0.01; *** *p* < 0.001). (**c**) Duration of sensory responses in the three groups under the co-administration regimen (*n* = 6; statistical significance as above). (**d**) Representative images of human dorsal skin from the control, P5-treated, and CPZ groups at 1 and 3 h post-capsaicin challenge (pre-administration regimen). (**e**) Sensory scores for the three groups in the pre-administration regimen (*n* = 6; statistical significance as above). (**f**) Duration of sensory responses in the three groups under the pre-administration regimen (*n* = 6; statistical significance as above). All statistical data are given as average ± SEM.

TRPV1 is expressed in sensory nerve endings and keratinocytes in human skin, where it serves as a key mediator of burning pain, erythema, and pruritus [5,25]. To evaluate the inhibitory effects of P5 on human TRPV1, we conducted a capsaicin-induced skin hypersensitivity test using a skin patch model in healthy volunteers. Two experimental paradigms were employed: simultaneous application, in which P5 and capsaicin were applied together, and pre-treatment, in which P5 was applied one hour before capsaicin. In the simultaneous application model, P5 significantly reduced capsaicin-induced pain, burning, and itching sensations compared with control, attenuated both the intensity and spread of erythema (Figure 4a,b), and delayed the onset of these discomforts (Figure 4c). In the pre-treatment model, P5, vehicle, or saline patches were applied to the inner forearm one hour before capsaicin exposure. P5 diminished subsequent erythema and pruritus (Figure 4d,e), but its ability to delay the onset of discomfort was limited compared with the simultaneous application model (Figure 4f). This reduced effect may be attributed to P5’s limited stability, with potential peptide degradation or loss of activity during the pre-treatment interval.

## 4. Discussion

Our workflow integrates a physics- and mathematics-driven pentapeptide library construction with a tiered SBVS cascade (RDKit → LigPrep conformer generation → Glide HTVS → Glide SP), enabling an efficient exploration of a chemically defined short-peptide space. This strategy offers three distinct advantages. First, the combinatorial, physics-based generation of peptide sequences produces a physically realistic library (SMILES → 3D conformers) rather than an abstract descriptor set, thereby preserving stereochemistry, backbone geometry, and side-chain rotamers that are critical for accurate docking and interaction mapping. Second, the HTVS→SP docking funnel provides a balance between efficiency and precision, rapidly triaging large conformational ensembles while allowing more exhaustive sampling of enriched subsets—an approach that has proven effective in both small-molecule and peptide virtual screens [26]. Third, structure-based peptide screening has now reached maturity; recent studies have reported successful discovery of bioactive peptides via docking and SBVS, underscoring the robustness of such pipelines for ion-channel targets [27,28].

Experimental validation further confirmed the strength of this framework. Although the virtual library contained sequences with no known biological activity a priori, five in silico hits (P1–P5) all showed measurable TRPV1 inhibition in electrophysiology assays. Among them, P5 emerged as the most potent candidate (IC_50_ ≈ 0.71 μM), combining favorable selectivity across key cardiac and neuronal safety channels with demonstrable anti-allergic efficacy in human skin assays. Collectively, these findings not only confirm the feasibility of mining random virtual libraries for bioactive peptides but also establish a practical “random-to-targeted” paradigm for ion-channel drug discovery. Importantly, our results suggest that even without prior optimization for drug-like properties, physics-driven virtual peptide libraries can yield biologically relevant leads. This methodological framework is therefore transferable: by expanding library size or applying target-specific modifications, similar pipelines may be applied to diverse ion channels and other membrane proteins, accelerating the discovery of novel peptide therapeutics.

Plug-like binding has been recognized as a classical pore-blocking mechanism for TRPV channel ligands. For instance, Ruthenium Red, a broad-spectrum cationic pore blocker, binds to sites near the filter region of TRPV channels and establishes a stable block through electrostatic interactions and hydrogen-bond networks. Cryo-EM studies of TRPV2 and TRPV5 have resolved its axial binding orientation within the pore, revealing a general “filter-proximal pore blockade” pattern [29]. In TRPV6, Ruthenium Red was directly visualized as a bottle-stopper–like plug embedded in the selectivity filter [30]. However, such plug-like blockade has not yet been structurally demonstrated in TRPV1, with only functional evidence suggesting that certain divalent cations, such as Ba^2+^ [31], and peptide toxins, such as AG-489 [32], may block the channel via external pore sites. Our docking results indicate that long-chain side chains of the pentapeptides P1–P5 penetrate the narrow filter pore to form directional hydrogen bonds with the GMGD filter sequence or adjacent residues. The remaining residues provide stabilizing interactions above the filter. This mode of interaction is highly consistent with reported pore-blocking mechanisms [29,30], suggesting that short peptides are also capable of producing stable TRPV1 inhibition through a plug-like mechanism. We further propose that the backbone carbonyls and acidic side chains of the selectivity filter act as natural anchoring points for hydrogen bonding. In addition, the funnel-shaped outer pore and the relatively short filter region provide sufficient buried surface area for peptide engagement. Moreover, additional interactions with outer pore loops and pore helices may extend ligand residence time and enhance binding selectivity.

A major limitation of linear all-L short peptides is their poor metabolic stability, with serum half-lives often ranging from only a few minutes to half an hour [33,34]. Given that P5 (DQKNC) is a linear L-pentapeptide, its in vivo stability is likely subject to similar constraints. Indeed, poor metabolic stability and short half-life remain major challenges for peptide-based TRPV1 inhibitors. Peptides are prone to proteolytic degradation [35,36], limiting their duration of action in biological fluids or tissues and often necessitating high or frequent dosing. However, in the case of P5, the topical route of administration turns this limitation into a potential advantage. Short peptides with fewer than 10 amino acids are generally more amenable to passive diffusion or facilitated transport across the stratum corneum [37]. Previous studies have shown that pentapeptides such as palmitoyl pentapeptide-4 and other cosmeceutical peptides can effectively penetrate the skin barrier and exert biological activity in dermal tissues [38,39]. Thus, despite its limited systemic stability, P5’s small size may facilitate effective skin permeation and local availability at epidermal and subepidermal sites where TRPV1 is expressed.

Consistent with this, our preliminary results indicate that P5 showed a pronounced inhibitory effect in the simultaneous application model, significantly reducing pruritus, erythema, and swelling. However, these findings should be considered preliminary rather than definitive evidence of clinical efficacy, given the limited sample size of the human study. In contrast, P5 displayed diminished efficacy in the pre-treatment model, which may be attributed to a combination of factors. (1) Partial degradation or diffusion during the pre-treatment interval may lower the effective concentration at the site of action. (2) P5 appears to bind more strongly to TRPV1 when the channel is activated by capsaicin, whereas its affinity for resting channels is lower [40]. (3) Pre-applied peptide may not sufficiently penetrate into subepidermal regions where TRPV1 is expressed, resulting in reduced local availability. Together, these findings suggest that while P5 is a potent and selective immediate TRPV1 blocker, further optimization of its stability and pharmacokinetic properties will be required to achieve preventive or sustained cutaneous anaphylaxis in preclinical development.

## 5. Limitations of the Study

Together, these findings suggest that while P5 is a potent and selective immediate TRPV1 blocker, further optimization of its stability and pharmacokinetic properties will be required to achieve preventive or sustained effects against cutaneous anaphylaxis in preclinical development.

Despite the robust integration of structure-based virtual screening and experimental validation, several limitations of this study should be acknowledged. First, although the virtual pentapeptide library was constructed using a physics- and mathematics-driven framework, its chemical diversity was still restricted to canonical L-amino acids and unmodified linear backbones. The absence of D-residues, non-natural amino acids, and backbone-cyclized scaffolds may limit both the chemical space coverage and metabolic stability of the identified peptides. Second, while docking to the TRPV1 outer pore pocket provided a plausible model for peptide interaction, no cryo-EM or mutagenesis experiments were performed to directly confirm the predicted binding mode. Third, the human skin patch test was conducted in a relatively small cohort, and in vivo animal data were not included, limiting statistical power and systemic evaluation. Finally, although the topical model demonstrated clear symptomatic relief, long-term safety, immunogenicity, and formulation stability under clinically relevant conditions were not assessed. Future work should therefore focus on expanding the virtual library to include D-residues and non-canonical amino acids to enhance metabolic stability and binding diversity, combining structural validation through cryo-EM to confirm pocket interactions, and systematically evaluating pharmacokinetics, safety, and formulation parameters in vivo. Addressing these aspects will be crucial for advancing P5 and related peptides toward clinical translation as next-generation TRPV1-targeted therapeutics.

## 6. Future Directions

Future studies will focus on lead optimisation and mechanistic refinement. Chemical stabilisation strategies, such as terminal modifications or incorporation of non-natural amino acids, will be explored to enhance peptide stability. In parallel, formulation approaches including liposomal encapsulation or penetration enhancers will be investigated to improve topical delivery and sustained local exposure. Structural and biophysical studies will be undertaken to validate peptide–TRPV1 binding modes. Importantly, the physics-driven random-to-targeted virtual screening framework established here is inherently transferable and will be extended to other ion channels and membrane proteins by expanding library size and incorporating target-specific constraints, thereby accelerating the discovery of novel peptide therapeutics.

## Data Availability

The raw dataset of the pentapeptide library has been deposited in Zenodo [https://doi.org/10.5281/zenodo.17410736] and is publicly available as of the publication date [41]. All original code has been deposited at Zenodo at [https://doi.org/10.5281/zenodo.17402501] and is publicly available as of the date of publication [42]. Any additional information required to reanalyze the data reported in this paper is available from the lead contact upon request. All data needed to evaluate the conclusions in the paper are present in the paper or the supplemental information. The plasmids can be provided by M.R. Further information and requests for resources and reagents should be directed to and will be fulfilled by, the Lead Contact, Mingqiang Rong (rongmq@hunnu.edu.cn).

## Supplementary Materials

The following supporting information can be downloaded at: https://www.mdpi.com/article/10.3390/cells15010079/s1, Figure S1: Representative binding modes of the five selected pentapeptides (P1–P5) docked to TRPV1, as obtained using Discovery Studio; Figure S2: UPLC/MS ion chromatogram of P1–P5; Table S1: Top 50 pentapeptides ranked by molecular docking score; Table S2: Capsazepine by molecular docking score; Table S3: Key ADMET Properties of the Top 20 Pentapeptide Candidates Predicted by SwissADME.
