# Supplementary material for: Virtual Screening–Guided Discovery of a Selective TRPV1 Pentapeptide Inhibitor with Topical Anti-Allergic Efficacy
Source: Cells. 2026 Jan 3;15(1):79. doi: 10.3390/cells15010079 (PMC12786063; doi:10.3390/cells15010079)
Supplement: Supplementary file 1 [file cells-15-00079-s001.zip › cells-4033375-supplementary.pdf]

Supplementary Table 1 Top 50 pentapeptides ranked by molecular docking score. Related to Figure 1.

Rank	Pentapeptide name	Sequence	Docking Score
1	P4	DNDWA	-10.609
2	P5	DQKNC	-10.119
3	P1	RHPKQ	-10.115
4	P2	RQWYC	-10.041
5	P3	RYYFR	-10.006
6	P6	DNSFC	-9.991
7	P7	NYFNC	-9.968
8	P8	RLNWC	-9.851
9	P9	DDIKN	-9.739
10	P10	RWWFC	-9.730
11	P11	RMWRQ	-9.719
12	P12	DFLNC	-9.706
13	P13	DQWNC	-9.663
14	P14	RFMWQ	-9.630
15	P15	NCINC	-9.609
16	P16	RIWLQ	-9.568
17	P17	DCYNC	-9.553
18	P18	DCFYC	-9.547
19	P19	RYWWD	-9.491
20	P20	RLAWC	-9.475
21	P21	RSWWN	-9.467
22	P22	RHHYC	-9.467
23	P23	RCRWQ	-9.457
24	P24	RQYPC	-9.455
25	P25	RYGWQ	-9.423
26	P26	RYLWQ	-9.408
27	P27	DCDFC	-9.402
28	P28	DSFFR	-9.395
29	P29	RQEDQ	-9.385
30	P30	NRQYQ	-9.378
31	P31	RWMMQ	-9.354
32	P32	CRFCN	-9.349
33	P33	RYFYD	-9.339
34	P34	RMFFR	-9.333
35	P35	RWWGC	-9.316
36	P36	RWRWC	-9.315
37	P37	DKGEC	-9.305
38	P38	RFKWC	-9.298
39	P39	RHKWQ	-9.297
40	P40	DCSNC	-9.287
41	P41	DWHNC	-9.285

42	P42	DNYSN	-9.278
43	P43	DHEWN	-9.235
44	P44	DDCVC	-9.207
45	P45	NVPNC	-9.195
46	P46	DQMKN	-9.191
47	P47	DCVFC	-9.186
48	P48	DNCDC	-9.167
49	P49	DYVNC	-9.166
50	P50	DSFYR	-9.144

Supplementary Table 2 Capsazepine by molecular docking score

Rank	Small molecule name	Docking Score
1	Capsazepine	-10.046

Supplementary Table 3. Key ADMET Properties of the Top 20 Pentapeptide Candidates Predicted by SwissADME

Pentapeptide name	H-bond acceptors	H-bond donors	Consensus Log P	ESOL Log S	GI absorption	BBB permeant
P1	11	11	-3.06	1.56	Low	No
P2	10	12	-1.2	-0.53	Low	No
P3	11	14	-0.27	-2.13	Low	No
P4	12	10	-2.97	1.33	Low	No
P5	12	10	-4.48	4.45	Low	No
P6	11	9	-2.89	1.55	Low	No
P7	11	9	-1.78	0.04	Low	No
P8	9	11	-1.48	0.37	Low	No
P9	13	10	-3.8	3.4	Low	No
P10	8	11	1.12	-3.23	Low	No
P11	10	14	-2.02	0.46	Low	No
P12	10	8	-1.27	-0.02	Low	No
P13	11	10	-2.84	1.03	Low	No
P14	9	11	-0.23	-1.16	Low	No
P15	9	8	-2.88	1.74	Low	No
P16	9	11	-0.22	-0.75	Low	No
P17	11	9	-2.68	0.97	Low	No
P18	10	8	-0.55	-1.29	Low	No
P19	11	13	0.18	-2.88	Low	No
P20	8	10	-0.4	-0.46	Low	No

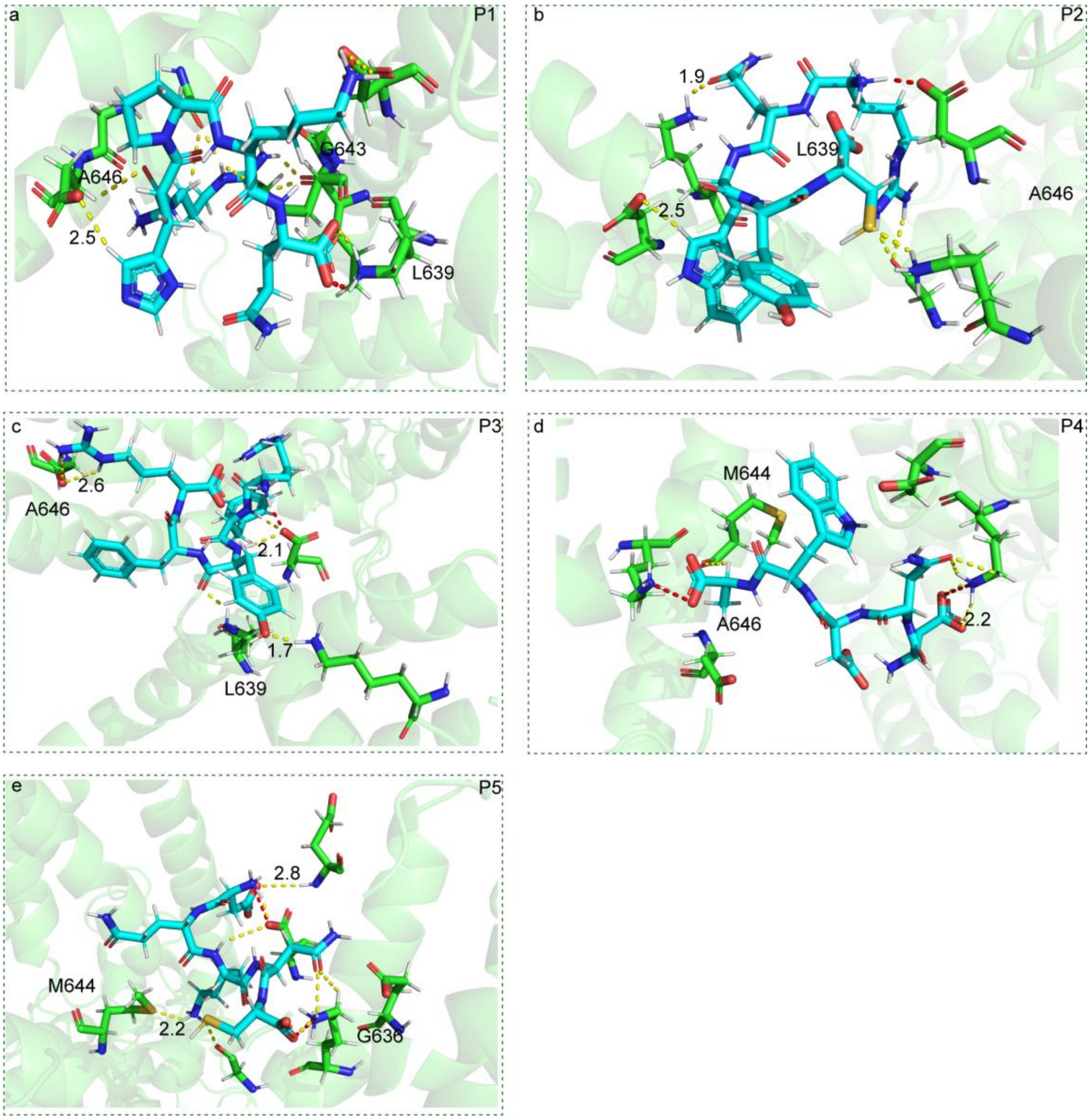

Supplementary Figure 1 (a–e) Representative binding modes of the five selected pentapeptides (P1–P5) docked to TRPV1, as obtained using Discovery Studio. Related to Figure 1.

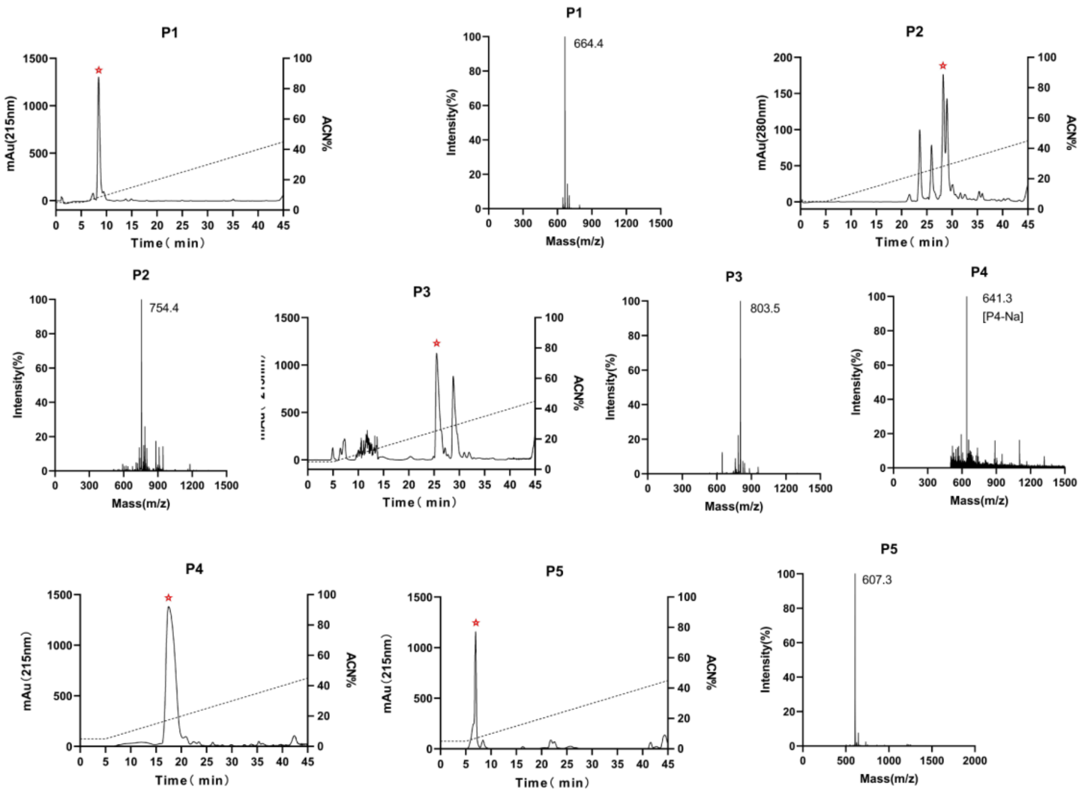

Supplementary Figure2 UPLC/MS ion chromatogram of P1-P5. Related to Figure 2.
